# The HSP70-fused foot-and-mouth disease epitope elicits cellular and humoral immunity and drives broad-spectrum protective efficacy

**DOI:** 10.1038/s41541-021-00304-9

**Published:** 2021-03-26

**Authors:** Hyundong Jo, Bong Yoon Kim, So Hui Park, Hyun Mi Kim, Sung Ho Shin, Seong Yun Hwang, Su-Mi Kim, Byounghan Kim, Jong-Hyeon Park, Min Ja Lee

**Affiliations:** 1 Animal and Plant Quarantine Agency, 177, Hyeoksin 8-ro, Gimcheon City, 39660 Gyeongsangbuk-do Republic of Korea; 2 Watson RnD Sharing Co. Ltd., 19, Sanmaru-ro, Guri-si, 11901 Gyeonggi-do Republic of Korea

**Keywords:** Inactivated vaccines, Infectious diseases, Adjuvants

## Abstract

Current foot-and-mouth disease (FMD) vaccines have significant limitations, including side effects due to oil emulsions at the vaccination site, a narrow spectrum of protective efficacy, and incomplete host defenses mediated by humoral immunity alone. To overcome these limitations, new FMD vaccines must ensure improved safety with non-oil-based adjuvants, a broad spectrum of host defenses within/between serotypes, and the simultaneous induction of cellular and humoral immunity. We designed a novel, immune-potent, recombinant protein rpHSP70-AD that induces robust cellular immunity and elicits a broad spectrum of host defenses against FMD virus (FMDV) infections. We demonstrated that an oil emulsion-free vaccine containing rpHSP70-AD mediates early, mid-term, and long-term immunity and drives potent host protection against FMDV type O and A, suggesting its potential as an FMD vaccine adjuvant in mice and pigs. These results suggest a key strategy for establishing next-generation FMD vaccines, including novel adjuvants.

## Introduction

Foot-and-mouth disease (FMD) mainly affects cloven-hooved animals such as cattle, pigs, sheep, goats, deer, and wild ruminants. Clinically, FMD causes blister formation on the lips, tongue, gums, nose, or limbs and severe illness due to a rapid increase in body temperature and loss of appetite. The disease can also lead to myocarditis and eventually death in young animals. FMD results in serious economic losses in the livestock industry due to its rapid propagation and the resulting loss of productivity^1,2^. FMD is classified as a highly contagious animal disease and is designated as a “disease to be managed” by the World Organization for Animal Health (Office International des Épizooties; OIE). As such, any cases of FMD outbreaks should be reported to the OIE^3^.

FMD virus (FMDV) is classified into seven different serotypes (A, O, C, Asia 1, SAT1, SAT2, and SAT3)^4^. Serotype O consistently occurs around the world, while serotype A and serotype Asia 1 sporadically occur in East Asia, including China and North Korea. FMDV shows a significant genetic/antigenic difference within/between serotypes, making vaccine-mediated cross-protection or cross-immunity difficult^5,6^.

Vaccination is used to control the disease in countries affected by FMD. Furthermore, vaccination plays an important role in establishing a contingency plan in FMD-free countries^7^. Worldwide, most FMD vaccines use the inactivated (killed) virus as the antigen, and their efficacies have been improved by using oil adjuvants (double oil emulsion or single oil emulsion). However, it takes a long time to attain an optimal antibody titer to achieve a certain level of defense after vaccination. In addition, pigs have lower antibody titers than cattle, shorter antibody persistence, low immunogenicity, and side effects at the site of vaccination, including the formation of granuloma, fibrosis, and abscesses due to the oil adjuvants. Other disadvantages, including safety issues and interference from maternally derived antibodies, have been reported^7–9^. Moreover, the FMD vaccine focuses on inducing a humoral immune response rather than a cellular immune response, but this defense is imperfect. Therefore, the development of new FMD vaccines with a simultaneous induction of a cellular and humoral immune response, broader spectrum of defense and minimal side effects is warranted. An ideal vaccine should overcome the limitations of the current commercially available vaccines, rapidly induce antibody titers post-vaccination and host defense in early stages of FMDV infection, maintain a high-titer of antibodies and long-lasting memory responses, provide complete protection, suppress viral shedding during FMDV infection, induce a broad spectrum of defenses within/between serotypes, elicit robust immune responses by stimulating both cellular and humoral immune responses, and overcome interferences from maternally derived antibodies.

Several studies related to adjuvants for improving the efficacy of FMD vaccines and host defenses against FMDV infection reported the use of receptor-specific immunostimulants; pattern recognition receptor (PRR) ligands such as R848, poly(I:C)^10^, muramyl dipeptide, monophosphoryl lipid, and β-glucan^11^, immune enhancers; rapeseed oil and ginseng root saponin^12^, and commercially available adjuvants; ISA 201, ISA 206, Emulsigen-D, and Carbigen^13^. However, complete immunity and optimal induction of host defenses have not yet been achieved.

Meanwhile, we proposed novel adjuvants optimized for cattle and pigs, especially adjuvants and FMD vaccine compositions that simultaneously enhance cellular and humoral immune responses by selecting PRR ligands as immunostimulants^14^. We reported the development of a recombinant immunopotent FMD vaccine strain and an advanced FMD vaccine platform by spiking the active sites of the immune-enhancing molecules HSP70 and T cell epitope 3A onto the surface of FMDV^15^.

Based on these findings, we synthesized an immunopotent recombinant protein, rpHSP70-AD, by combining: (1) An immuno-active molecule such as *hsp70* (linker of innate and adaptive immunity); (2) immune cell epitope of FMDV such as (i) 3A (universal T cell epitope of FMDV), (ii) invasion (exogenous T cell epitope), (iii) B cell epitope of FMDV type O (O/JC/SKR/2014, O/TWN/97), and (iv) B cell epitope of FMDV type A (A/GP/2018, A/GVII:Ban-GA); (3) the VP1 region of FMDV; (4) a delivery molecule such as astrotactin 1-derived peptide (AP, novel transdermal delivery peptide); (5) an immune-enhancing peptide such as Pan HLA-DR reactive epitope (PADRE); and (6) the “GGSGG” amino acid sequence as a linker to produce a potent FMD vaccine adjuvant. Thus, a novel vaccine composition containing rpHSP70-AD as an active adjuvant for preventing FMD was developed. Here, we demonstrate that this new recombinant protein, rpHSP70-AD, can ameliorate the side effects induced by oil emulsion, act as an antigen in the host to induce a broad spectrum of defenses, protect the host in the early stage of viral infection, play a pivotal role as an immune-enhancing adjuvant that simultaneously induces strong cellular and humoral immune responses, and effectively induce early, mid-term, and long-term immunity.

## Results

### The novel immunopotent recombinant protein rpHSP70-AD effectively stimulates inflammatory cytokine expression in vivo, inducing a significant cellular immune response

**Fig. 1:**
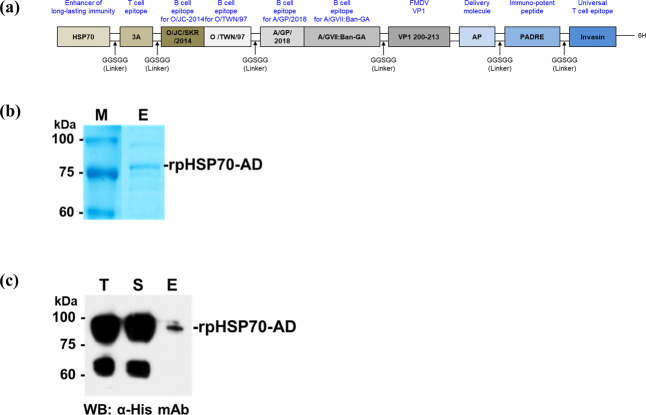
A schematic diagram of the novel immunopotent porcine recombinant protein, rpHSP70-AD, and expression of rpHSP70-AD. Schematic diagram of the novel immunopotent porcine recombinant protein, rpHSP70-AD (**a**); The novel immunopotent recombinant protein rpHSP70-AD combines the multiple indicated active immunopotent domains. The detailed strategy is described in Methods, and the sequence information for each molecule is described in Supplementary Table 1. Sun-Gel-stained SDS-PAGE gel of rpHSP70-AD, M; molecular size marker, E; purified elution fraction (**b**); Western blot of purified recombinant rpHSP70-AD, T; total cell lysates, S; soluble fraction of cell lysates, E; purified elution fraction (**c**).

**Fig. 2:**
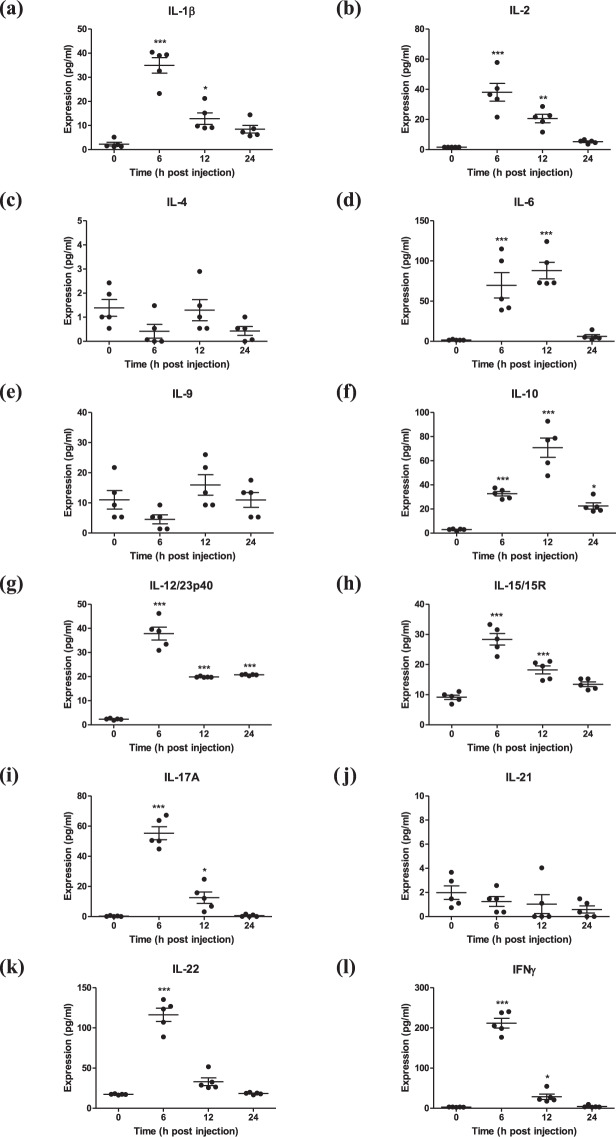
rpHSP70-AD mediated cytokine expression and temporal kinetics in vivo. C57BL/6 mice (*n* = 5/group/time point) were injected intraperitoneally with 50 μg rpHSP70-AD and sacrificed at 0, 6, 12, and 24 h post-injection. Peritoneal lavage fluid was collected by washing the peritoneal cavity with 2 mL chilled DPBS. Samples were then centrifuged, and the supernatant was analyzed for cytokines using ELISA. Cytokine profiles and temporal kinetics in vivo induced by rpHSP70-AD: IL-1β (**a**); IL-2 (**b**); IL-4 (**c**); IL-6 (**d**); IL-9 (**e**); IL-10 (**f**); IL-12/23p40 (**g**); IL-15/15 R (**h**); IL-17A (**i**); IL-21 (**j**); IL-22 (**k**); IFNγ (**l**). The data are represented as the mean ± SEM of triplicate measurements (*n* = 5). Statistical analyses were performed using one-way ANOVA followed by Tukey’s post hoc test. ^*^p < 0.05, ^***^p < 0.001.

A schematic diagram of the development of rpHSP70-AD is shown in Fig. 1a and Supplementary Table S1. The solubilized and purified rpHSP70-AD recombinant protein was observed in the purified fraction of SDS-PAGE (Fig. 1b and Supplementary Fig. S1a) and western blot analysis using an anti-His antibody (Fig. 1c and Supplementary Fig. S1b). To evaluate the effect of rpHSP70-AD on systemic immune response and cellular immunity, rpHSP70-AD was intraperitoneally injected into mice. Then, cytokine profiles and temporal kinetics were analyzed in mouse peritoneal lavage fluid (Fig. 2a–l). Six hours after rpHSP70-AD injection, the expression of proinflammatory cytokines IL-1β (Fig. 2a), IL-2 (Fig. 2b), IL-12/23p40 (Fig. 2g), IL-15/15R (Fig. 2h), IL-17A (Fig. 2i), IL-22 (Fig. 2k), and IFNγ (Fig. 2l) significantly increased (*p* < 0.001) and then decreased (IL-Iβ: *p* < 0.05; IL-2: *p* < 0.01; IL-12/23p40: *p* < 0.001; IL-15/15 R: *p* < 0.001; IL-17A: *p* < 0.05; IFNγ: *p* < 0.05). The expression of IL-6 (Fig. 2d) was significantly higher after 6 and 12 h (*p* < 0.001). However, the expression of the anti-inflammatory cytokine IL-10 (Fig. 2f) was the highest after 12 h (*p* < 0.001) and decreased in 24 h (*p* < 0.05). The expression of IL-4 (Fig. 2c), IL-9 (Fig. 2e), and IL-21 (Fig. 2j) was very low, and no significant differences were observed.

### rpHSP70-AD induces a broad spectrum of host defenses, indicating a strong protective effect against FMDV type O and A infections

**Fig. 3:**
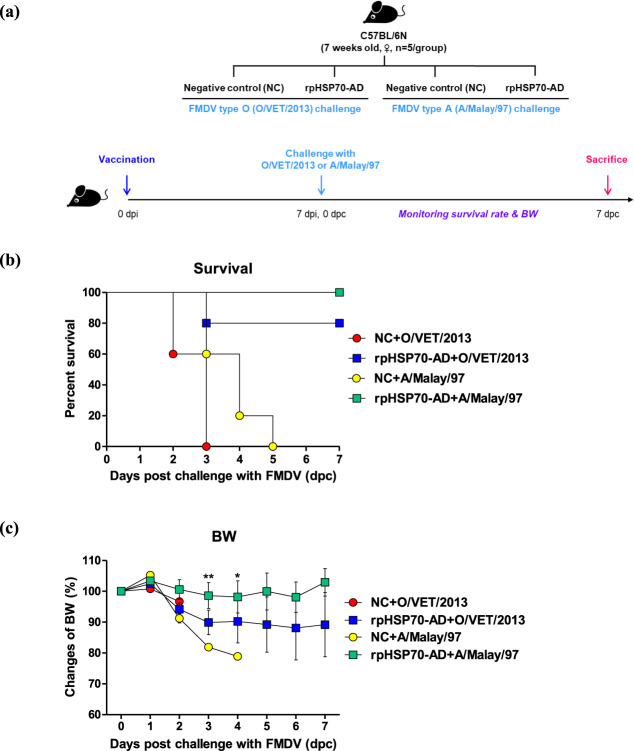
rpHSP70-AD induced a broad spectrum of host protection against FMDV O (O/VET/2013) and FMDV A (A/Malay/97) infection in mice. C57BL/6 mice (*n* = 5/group) were administered with test vaccine including 10 μg rpHSP70-AD, ISA 206 (oil-based emulsion, 50%, w/w), 10% Al(OH)_3_, and 15 μg Quil-A. A negative control (NC) group was injected with the same volume of PBS. The test vaccines were injected intramuscularly into mice that were later challenged with FMDV O (100 LD_50_ O/VET/2013) and FMDV A (100 LD_50_ A/Malay/97) at 7 days post-vaccination. The survival rates and body weights were monitored for 7 days post-challenge. Experimental strategy (**a**); survival rates post-challenge with O/VET/2013 and A/Malay/97 (**b**); changes in body weight post-challenge with O/VET/2013 and A/Malay/97 (**c**). The data represent the mean ± SEM of triplicate measurements (*n* = 5/group). Statistical analyses were performed using two-way ANOVA with Bonferroni correction. ^*^p < 0.05; ^**^p < 0.01.

The rpHSP70-AD-mediated host defense against a broad range of FMDVs was evaluated in mice (Fig. 3a–c). The recombinant immunopotent protein, rpHSP70-AD, contains a part of the VP1 sequence, which has the strongest immunogenicity in the FMD viral sequence, and the B cell epitope of FMDV type O and A that would act as an antigen. The antigen was excluded during vaccine preparation (without antigen), and 100 μL test vaccine containing rpHSP70-AD was injected into the mouse thigh. Seven days post-vaccination (dpv), mice were challenged with FMDV type O (100 LD_50_ O/VET/2013, ME-SA topotype) and FMDV type A (100 LD_50_ A/Malay/97, Asia topotype). The survival rate and bodyweight changes were monitored for 7 days post-challenge (dpc) (Fig. 3a). The group vaccinated with rpHSP70-AD showed 80 and 100% protection against O/VET/2013 and A/Malay/97, respectively (Fig. 3b). One hundred percent of the negative control (NC) groups challenged with the type O and A FMDVs died at 3 and 5 dpc, respectively. Less than 10% weight loss was observed in the vaccine group challenged with O/VET/2013, whereas no weight loss was observed in the vaccine group challenged with A/Malay/97 (Fig. 3c).

### Oil emulsion-free vaccines containing the rpHSP70-AD adjuvant elicit strong early, mid-term, and long-term immunity and memory responses in mice

**Fig. 4:**
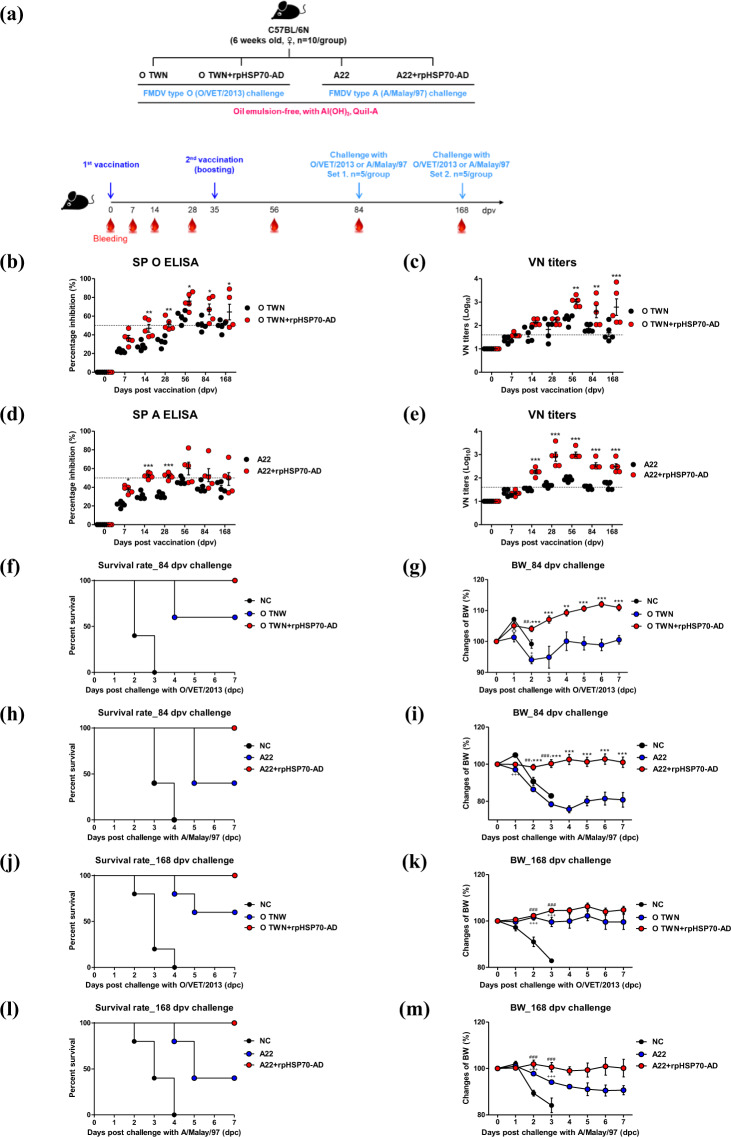
rpHSP70-AD mediated early, mid-term, and long-term immune responses and host defense against FMDV infection in mice. C57BL/6 mice (*n* = 5/group) were administered oil emulsion-free test vaccines, including O TWN antigen, A22 antigen, or combined O TWN and A22 antigens with rpHSP70-AD. The positive control received 1.5 μg (1/10 dose for cattle and pig use) O TWN antigen or A22 antigen without (w/o) oil emulsion, 10% Al(OH)_3_, and 15 μg Quil-A. The negative control received the same volume of PBS. Vaccination was performed twice at 35-day intervals. Study strategy (**a**); antibody titers by SP O ELISA (**b**); O/TWN/97 VN titers (**c**); antibody titers by SP A ELISA (**d**); A22/IRAQ VN titers (**e**); survival rate at 84 days post-vaccination challenge with FMDV O (O/VET/2013) (**f**); changes in body weight at 84 days post-vaccination challenge with FMDV O (O/VET/2013) (**g**); survival rate at 84 days post-vaccination challenge with FMDV A (A/Malay/97) (**h**); changes in body weight at 84 days post-vaccination challenge with FMDV A (A/Malay/97) (**i**); survival rate at 168 days post-vaccination challenge with FMDV O (O/VET/2013) (**j**); changes in body weight at 168 days post-vaccination challenge with FMDV O (O/VET/2013) (**k**); survival rate at 168 days post-vaccination challenge with FMDV A (A/Malay/97) (**l**); changes in body weight at 168 days post-vaccination challenge with FMDV A (A/Malay/97) (**m**). The data represent the mean ± SEM of triplicate measurements (*n* = 5/group). Statistical analyses involved two-way ANOVA with Bonferroni correction. ^+^*p* < 0.05; ^++^*p* < 0.01; and ^+++^*p* < 0.001: NC vs PC (O TWN- or A22-only group); ^#^*p* < 0.05; ^##^*p* < 0.01; and ^###^*p* < 0.001: NC vs Exp. (O TWN + rpHSP70-AD or A22 + rpHSP70-AD group); ^*^p < 0.05; ^**^p < 0.01; and ^***^p < 0.001: PC vs Exp.

To overcome the difficulties of rapid antigen release and the maintenance of high-titer antibodies in an oil emulsion-free state and evaluate the efficacy of the new recombinant rpHSP70-AD adjuvant, especially as an immunostimulant, an oil emulsion-free rpHSP70-AD vaccine was prepared, and early, mid-term, and long-term immunity and memory responses were observed in mice (Fig. 4a–m).

Mice were vaccinated with a test vaccine and administered a booster dose on 35 dpv. Blood was collected on 0, 7, 14, 28, 56, 84, and 168 dpv (Fig. 4a) and used for serological analysis, including structural protein (SP) O ELISA (Fig. 4b), SP A ELISA (Fig. 4d), and virus-neutralizing (VN) titers (Fig. 4c, e). Mice were challenged with FMDV (100 LD_50_ of O/VET/2013, ME-SA topotype or A/Malay/97, Asia topotype) on 84 and 168 dpv. Survival rates (Fig. 4f, h, j, l) and bodyweight changes (Fig. 4g, i, k, m) were monitored up to 7 dpc.

The group vaccinated with O/TWN/97-R (O TWN) + rpHSP70-AD showed significantly higher antibody titers in SP O ELISA (Fig. 4b) than the group vaccinated with O TWN (without rpHSP70-AD) at 14 and 28 dpv (*p* < 0.01). Antibody titers were maintained for up to 168 days after the booster vaccination (*p* < 0.05). In the case of VN titers (Fig. 4c), the group vaccinated with O TWN + rpHSP70-AD showed higher levels than the O TWN-vaccinated group, but the difference was not significant. After the booster vaccination, the titer level of the O TWN + rpHSP70-AD group was significantly higher from 56 to 168 dpv (*p* < 0.01, *p* < 0.001).

The antibody titer was also analyzed via SP A ELISA (Fig. 4d); it was significantly higher in the A22/IRAQ-R (A22) + rpHSP70-AD group than in the A22-only group (without rpHSP70-AD) vaccinated 7 days after the primary vaccination (*p* < 0.05); it was also significantly higher at 14 and 28 dpv (*p* < 0.001). No significant difference was observed between the two groups after boosting. Additionally, the A22 + rpHSP70-AD group showed continuously higher VN titers (Fig. 4e) than the A22-only group (*p* < 0.001) between 14 and 168 days after primary vaccination.

The FMDV (100 LD_50_ O/VET/2013, ME-SA topotype or 100 LD_50_ A/Malay/97, Asia topotype) challenge experiment showed a 100% survival rate for both the O TWN + rpHSP70-AD and A22 + rpHSP70-AD groups against FMDV O and FMDV A infections at 84 dpv (Fig. 4f, h). There were no weight changes in either group (Fig. 4g, i). In addition, 100% survival (Fig. 4j, l) was observed at 168 dpv, with negligible weight changes (Fig. 4k, m). Meanwhile, the O TWN group and A22 group showed a 60% and 40% defense rate, respectively, when challenged with FMDV O type (O/VET/2013) and FMDV A type (A/Malay/97) at 84 and 168 dpv, respectively.

### rpHSP70-AD promotes cell proliferation and triggers cellular immune responses in porcine peripheral blood mononuclear cells (PBMCs)

**Fig. 5:**
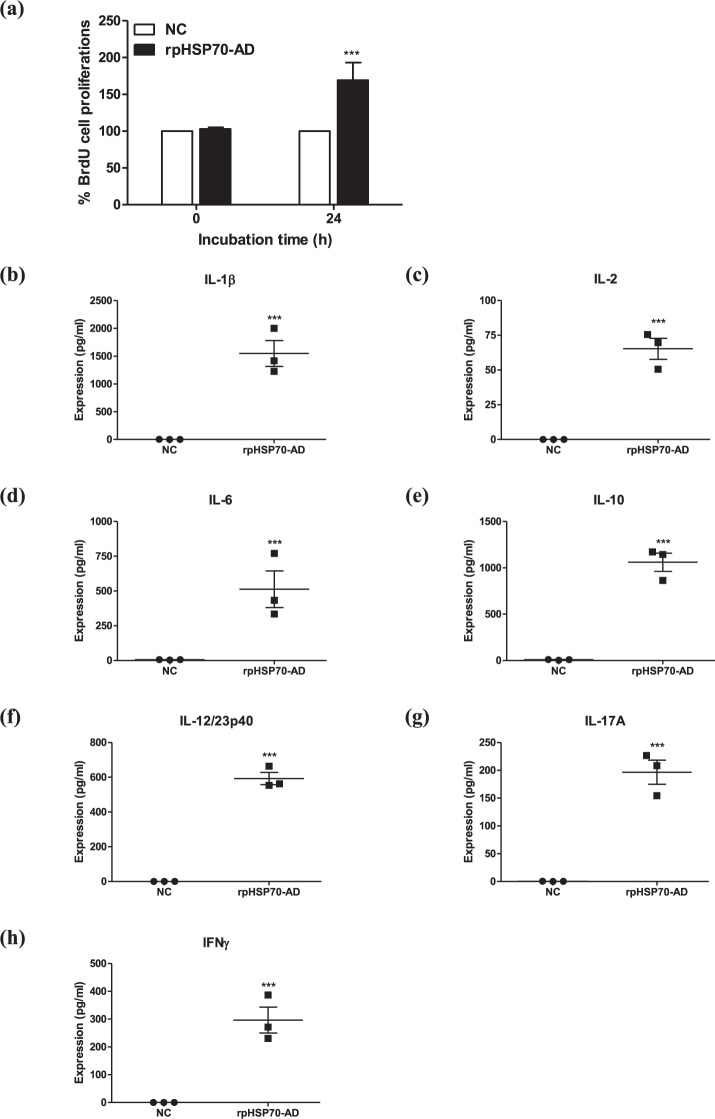
rpHSP70-AD induced porcine PBMC cell proliferation and cytokine expression. Porcine PBMCs were co-incubated with rpHSP70-AD (final concentration: 2 μg/mL). Exactly 0 and 24 h after co-incubation, cell proliferation was tested using the BrdU ELISA kit. Cell culture supernatants were harvested for cytokine ELISA. In vitro cell proliferation induced by rpHSP70-AD in porcine PBMCs (**a**); IL-1β (**b**); IL-2 (**c**); IL-6 (**d**); IL-10 (**e**); IL-12/23p40 (**f**); IL-17A (**g**); and IFNγ (**h**) expression induced by rpHSP70-AD in porcine PBMCs. The data are represented as the mean ± SEM of triplicate measurements (*n* = 3). Statistical analyses were performed using unpaired two-tailed Student’s *t*-test. ^***^p < 0.001.

Before the in vivo experiment using target animals (pigs), naïve PBMCs were individually isolated from the whole blood of FMD antibody-seronegative pigs (*n* = 3/group). The PBMCs were treated with rpHSP70-AD, and cellular immune responses, including cell proliferation and inflammatory cytokine expression, were evaluated (Fig. 5a–h). Cell proliferation (BrdU incorporation assay) increased by 169.23 ± 24.08% upon 24 h treatment with rpHSP70-AD, compared to the NC group (*p* < 0.001, Fig. 5a). ELISA assays of porcine cytokine expression showed that rpHSP70-AD treatment significantly upregulated the proinflammatory cytokines IL-1β (Fig. 5b), IL-2 (Fig. 5c), IL-6 (Fig. 5d), IL-12/23p40 (Fig. 5f), IL-17A (Fig. 5g), and IFNγ (Fig. 5h) after 24 h (*p* < 0.001). Furthermore, the expression of the anti-inflammatory cytokine IL-10 (Fig. 5e) was significantly higher upon rpHSP70-AD treatment than in the NC group at 24 h (*p* < 0.001).

### An oil emulsion-free test vaccine including the rpHSP70-AD adjuvant simultaneously elicits a potent cellular and humoral immune response in pigs

**Fig. 6:**
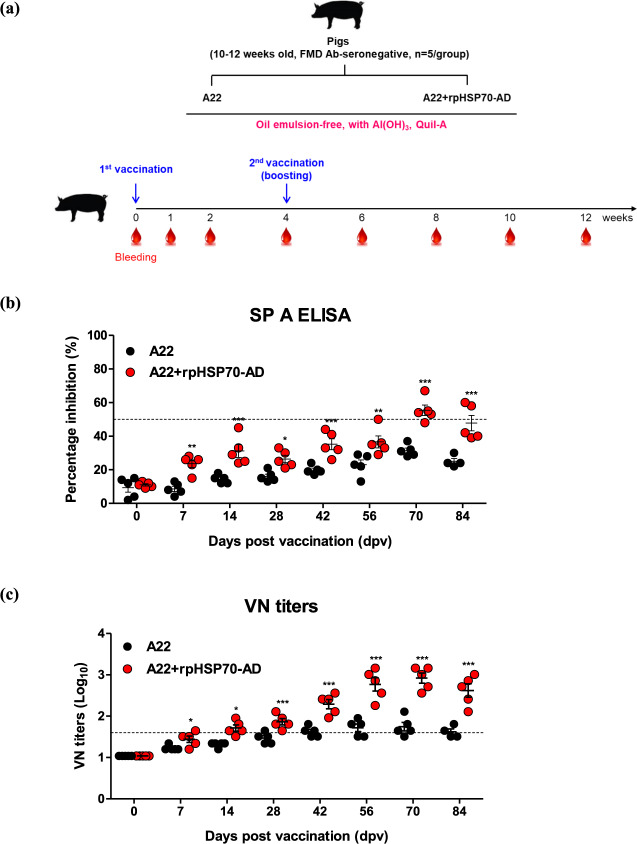
rpHSP70-AD mediates early, mid-term, and long-term immune responses in pigs. For pig experiments, FMD antibody-seronegative animals (10–12 weeks old) were used. Pigs were divided into two groups (*n* = 5/group) and administrated oil emulsion-free test vaccines, including A22 antigen alone, or combined A22 antigens with rpHSP70-AD. The positive control group received 15 μg (one dose for cattle and pig use) A22 antigen without (w/o) oil emulsion, 10% Al(OH)_3_, and 150 μg Quil-A. The vaccination was performed twice at 28-day intervals, with 1 mL vaccine (one dose) injected via a deep intramuscular route on the animals’ necks. Blood samples were collected at 0, 7, 14, 28, 42, 56, 70, and 84 days post-vaccination in pigs for serological assays. Study strategy (**a**); SP A antibody titers (**b**); and A22/IRAQ VN titers (**c**). The data represent the mean ± SEM of triplicate measurements (*n* = 5/group). Statistical analyses were performed using two-way ANOVA with Bonferroni correction. ^*^p < 0.05; ^**^p < 0.01; and ^***^p < 0.001.

To establish the effect of rpHSP70-AD-mediated cellular and humoral immunity in pigs, we used an oil emulsion-free test vaccine containing rpHSP70-AD as an adjuvant and evaluated test vaccine-mediated early, mid-term, and long-term immunity and memory responses. FMD antibody-seronegative pigs were vaccinated, and a booster vaccine was administered on 28 dpv. Blood was collected on 0, 7, 14, 28, 42, 56, 70, and 84 dpv, and serological analysis was performed (Fig. 6a).

The antibody titers via SP A ELISA were significantly higher in the A22 + rpHSP70-AD group during the initial vaccination period, and at 7 and 14 dpv, than in the A22-only group (*p* < 0.05, *p* < 0.01). After receiving the booster vaccine on 28 dpv, antibodies were significantly higher in the A22 + rpHSP70-AD group until 84 dpv (*p* < 0.01, *p* < 0.001) (Fig. 6b). VN titers using A22/IRAQ, a homologous virus for the A22 antigen, showed similar results to SP A ELISA and were significantly higher in the A22 + rpHSP70-AD group until 14 dpv (*p* < 0.05). After receiving the booster vaccine on 28 dpv, VN titers were significantly higher than the control group (A22 group) from 42 to 84 dpv (*p* < 0.001) (Fig. 6c).

### Single-shot of an oil emulsion-free test vaccine including rpHSP70-AD as an adjuvant drives potent host protection against FMDV type O and FMDV type A infection in pigs

To evaluate the effect of rpHSP70-AD-mediated host defense in pigs, FMD antibody-seronegative animals were immunized with a single dose of an oil emulsion-free bivalent (O + A; O BE + A YC antigen) test vaccine with or without (w/o) rpHSP70-AD as an adjuvant and challenged with FMDV type O (O/SKR/JC/2014, Asia topotype) or FMDV type A (A/SKR/GP/2018, Asia topotype) at 28 dpv. The NC group was treated with an equal volume of PBS and challenged with FMDV type O or FMDV type A following the same schedule.

**Fig. 7:**
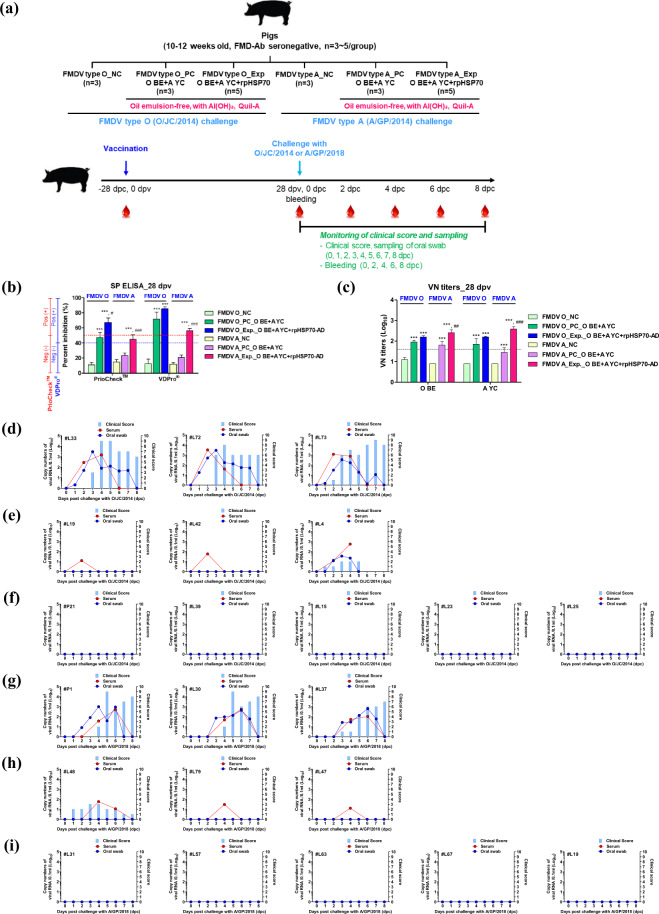
rpHSP70-AD mediates host defense against FMDV O and A infection in pigs. For the challenge experiments, pigs (10–12 weeks old, *n* = 3–5/group) were administered oil emulsion-free test vaccines, including O BE + A YC antigen alone or combined O BE + A YC antigens with rpHSP70-AD. Vaccinated pigs were challenged with FMDV type O (O/SKR/JC/2014) or FMDV type A (A/SKR/GP/2018) on the heel bulb at 10^5^ TCID_50_/100 μL. Study strategy (**a**); SP O and SP A antibody titers (**b**); O/SKR/BE/2017 and A/SKR/YC/2017 VN titers (**c**); clinical score and the amount of virus in serum and oral swab from negative control (*n* = 3/group) pigs against FMDV type O (O/SKR/JC/2014) (**d**) or FMDV type A (A/SKR/GP/2018) (**g**); clinical score and the amount of virus in serum and oral swab from positive control (O BE + A YC, *n* = 3/group) pigs against FMDV type O (O/SKR/JC/2014) (**e**) or FMDV type A (A/SKR/GP/2018) (**h**); clinical score and the amount of virus in serum and oral swab from experimental group (O BE + A YC + rpHSP70-AD, *n* = 5/group) pigs against FMDV type O (O/SKR/JC/2014) (**f**) or FMDV type A (A/SKR/GP/2018) (**i**). The left *Y*-axis of the graph shows the amount of virus in sera and oral swab as log_10_ values; the right Y-axis shows the clinical index as the maximum value of 10 points. The data represent the mean ± SEM of triplicate measurements (*n* = 3∼5/group). Statistical analyses involved two-way ANOVA with Bonferroni correction. ^***^p < 0.001: NC vs PC, NC vs Exp.; ^#^*p* < 0.05; ^##^*p* < 0.01; ^###^*p* < 0.001: PC vs Exp.

Considering the specificity of the antigen-coated SP ELISA kit, the antibody titer was double-checked using the PrioCHECK^TM^ kit and VDPro^®^ kit. The antibody titers by SP O ELISA and SP A ELISA using the PrioCHECK^TM^ kit at 28 dpv were significantly higher in the O BE + A YC + rpHSP70-AD group than in the O BE + A YC group (*p* < 0.05 for type O or *p* < 0.001 for type A) (Fig. 7b). Although no significance was observed in the antibody titers by SP O ELISA using the VDPro^®^ kit at 28 dpv between the O BE + A YC + rpHSP70-AD group and the O BE + A YC group, it was higher in the O BE + A YC + rpHSP70-AD group than in the O BE + A YC group. The antibody titers established by SP A ELISA using the VDPro^®^ kit were significantly higher in the O BE + A YC + rpHSP70-AD group than in the O BE + A YC group (*p* < 0.001).

VN titers using O/SKR/BE/2017 and A/SKR/YC/2017, a homologous virus for the O BE and A YC antigens, were similar to those of antibody titers by SP O and SP A ELISA. Although no significance was observed in the VN titers for O/SKR/BE/2017 between the O BE + A YC + rpHSP70-AD and O BE + A YC groups at 28 dpv, VN titers for A/SKR/YC/2017 were significantly higher in the O BE + A YC + rpHSP70-AD group than in the O BE + A YC group (*p* < 0.001).

Following the challenge, several FMD parameters were analyzed, including clinical signs, viremia in sera, and oral swab. The NC group (animals #33, #72, and #73 against FMDV type O infection; animals #P1, #L30, and #L37 against FMDV type A infection) showed 100% of the typical clinical symptoms of FMD, and a high level of virus was detected in sera and oral swab (Fig. 7d, g, Supplementary Fig. S2a, g). In the positive control group, one out of three animals showed clinical signs of FMD against FMDV type O (animal #L4) or FMDV type A (animal #L48) infection, designating partial protection. Although virus release from oral swabs was not observed, the viremia from sera was detected between 1–3 (log_10_) for FMDV type O infection and 1–2 (log_10_) for FMDV type A infection (Fig. 7e, h, Supplementary Fig. S2b, h).

Contrastingly, the animals in the O BE + A YC + rpHSP70-AD group (animals #P21, #L39, #L15, #L23, and #L25 against FMDV type O infection; animals #L31, #L57, #L63, #L67, and #L19 against FMDV type A infection) did not show any clinical signs of FMD, and there was no virus release from sera and oral cavities (Fig. 7f, i, Supplementary Fig. S2c, i), indicating 100% host protection.

## Discussion

Due to the recent occurrence of COVID-19 worldwide, interest in positive-strand RNA viruses [(+) RNA viruses] is increasing. (+) RNA viruses account for more than one-third of all viruses, including severe acute respiratory syndrome coronavirus (SARS-CoV), Middle East respiratory syndrome coronavirus, hepatitis C virus, West Nile virus, dengue virus, encephalomyocarditis virus, and FMDV. Among these, FMDV is a critical pathogen in animals with a great economic impact. It takes a long time for vaccines to be developed after the initial outbreak of these diseases, and achieving control of these viruses is difficult as genetic variation within each virus is diverse and develops fast^16,17^.

FMD is classified as an acute contagious disease, especially in pigs and cattle. Its rapid propagation and high mortality rate in young animals causes serious economic losses in the livestock industry^1,18^. Therefore, a vaccination policy using inactivated FMDV antigen is used to control FMD, but the FMD vaccine does not provide cross-protection against other serotypes. Thus, it is crucial to develop an FMD vaccine that can provide a wide range of defenses.

In the case of commercialized vaccines, classic immunopotentiators such as oil emulsions (Marcol 52, ISA 206, and ISA 50) and aluminum gel (Al(OH)_3_) are used as vaccine components to enhance antigen immunogenicity, but the exact mechanism underlying the induction of an immune response has not been revealed. Commercial FMD vaccines, containing conventional adjuvants, take a long period to induce antibody production and reach effective levels (induction of a humoral immune response) for host defense. Furthermore, commercial FMD vaccines show low antibody titers in pigs than in cattle, and despite high antibody titers, the defense against viral infection is not perfect^7–9^.

Notably, localized side effects at the vaccination site due to oil emulsions lead to great economic loss for pig farmhouses in Korea^19^. Despite the limitations of existing adjuvants, strict requirements for vaccine safety pose a major challenge in the successful development of new adjuvants. Thus, oil emulsions and aluminum gel remain the gold standard for all adjuvants^20^.

Considering previous studies, we performed an intensive study that used PRR ligands and cytokines as new FMD vaccine adjuvants to simultaneously enhance cellular and humoral immune responses in target animal species (cattle and pigs)^14^. In addition, we inserted 3A, a universal T cell epitope, and HSP70, which acts as a linker for innate and adaptive immune responses as “chaperokines” to the FMDV VP1 region to develop immunopotent FMD vaccine strains that stimulate strong, simultaneous cellular and humoral immune responses in mice and pigs^15^. Based on these findings, we developed a new porcine recombinant protein, rpHSP70-AD (Fig. 1, Supplementary Table S1 and Supplementary Fig. S1), which simultaneously induces potent cellular and humoral immune responses and exhibits a broad spectrum of defense against FMDV O type and FMDV A type.

Among the structural proteins of FMDV, VP1 or a fragment of this capsid protein is known to induce neutralizing antibodies in animals^21^. Although there are reports that antigenic sites are also present in VP2^22^ and VP3^23^, the major antigenic sites and B cell epitopes of FMDV have been identified as 1) VP1 (140–160) and 2) VP1 (200–213), at the extreme carboxy terminus of the protein. Based on this background, the VP1 of FMDV was selected for the design of the immunopotent protein in the present this study. In addition, HSP70 has the largest amino acid size and is the main component of rpHSP70-AD; therefore, the active site of HSP70 was designed to be located at the N-terminus of the protein structure, so that the first molecule could successfully be processed and the inserted molecule could be expressed sequentially.

We then evaluated the potential of rpHSP70-AD as a novel adjuvant to enhance early, mid-term, and long-term immunity and memory responses in mice and pigs. The peritoneum is a very useful source to evaluate systemic immune responses. Peritoneal exudate cells include innate immune cells such as antigen-presenting cells (APCs), including dendritic cells, macrophages (MΦs), and monocytes, and unconventional T cells, including gamma delta (γδ) T cells, invariant natural killer (iNKT) T cells, and mucosal-associated invariant T cells. Therefore, peritoneal exudate is suitable for studying cellular immune responses. Our in vivo study in mice showed a high level of proinflammatory cytokine expression, including IL-1β, IL-2, IL-6, IL-12/23p40, IL-15/15R, IL-17A, IL-22, and IFNγ, 6 h after intraperitoneal (i.p.) injection of rpHSP70-AD. The expression of the anti-inflammatory cytokine IL-10 reached its maximum at 12 h after the injection (Fig. 2). The temporal kinetics related to the expression of these cytokines are interpreted as a mechanism to maintain host homeostasis in vivo and control autoimmune diseases via persistent inflammatory responses. IFNγ is a key modulator of the T cell-mediated cellular immune response^24^, and IFNγ-induced immunomodulatory effects include immune cell activation, trafficking, differentiation, and direct intracellular antiviral activities^25^. IFNγ, which is mainly produced by natural killer cells, CD4^+^ T cells, and CD8^+^ T cells, induces IFN-stimulated genes and regulates immune response to viral infections^26^.

γδ T cells and iNKT cells rapidly produce IL-17A in response to stimulation by various pathogens such as bacteria, fungi, parasites, and viruses. Th17 cells are a major source of IL-17A. Importantly, IL-17A defends the host by recruiting neutrophils from mucosal- and non-mucosal tissues to the infection site in the early stages of infection, which subsequently clears the pathogens^27^. In addition, they activate APCs during viral infection, produce Th1^28^ and CTL^29^ responses, and play a key role in regulating B cell responses^30^.

In this study, the initiation of a fast and strong cellular immune response in the host by rpHSP70-AD was expected to contribute to host defense by effectively clearing the virus in the early stage of infection during the actual FMDV infection. Therefore, regardless of the inactivated FMDV (antigen)-mediated immune response, we evaluated the efficacy of test vaccines containing rpHSP70-AD without antigens to verify the host defense against a broad spectrum of FMDV infections by potentiating the rpHSP70-AD-induced cellular immune response. The administration of the test vaccine containing rpHSP70-AD without antigens showed enhanced host defense against FMDV O (O/VET/2013) and FMDV A (A/Malay/97) challenges (Fig. 3), which might be due to the following: (1) The powerful cellular immune response induced by rpHSP70-AD, which contains an HSP70 active site and acts as a “chaperokine”; (2) effective T and B cell stimulation due to FMDV T cell and B cell epitopes contained in the rpHSP70-AD amino acid sequence; (3) rpHSP70-AD acting as an antigen due to the VP1 sequence, which has the strongest immunogenicity among FMDVs; and (4) the presence of the immune-enhancing peptide, PADRE and a delivery molecule, AP.

Peptide vaccine studies using FMDV T cell and B cell epitopes^31–34^ have been actively conducted. More studies have attempted to synthesize recombinant proteins using multiple FMDV epitopes and use these proteins as vaccine candidates^35^. Previous studies on peptide and recombinant protein vaccines showed significant increases in antibody levels and neutralizing antibody titers in the serum. Further, these candidate vaccines showed the induction of considerable cellular responses, but they have not shown complete host protection against FMDV infection. This phenomenon in previous subunit vaccine studies may have two main possibilities. First, from the viewpoint of the FMDV vaccine antigen, these subunit vaccines were designed based only on the specific VP1 epitope of FMDV, so it was interpreted that the immunogenicity- and immune response-inducing effects in target animals would be lower than those of inactivated viral antigens. Second, in terms of the adjuvant, since several subunit vaccines are designed based on epitopes of specific cytokines or chemokines, which have immune-enhancing effects, it was interpreted that they would show incomplete immune responses within the host by promoting only the expression of the inserted molecule, and not multi-functional immunity. Based on these possibilities, to overcome the limitations of the previously studied subunit vaccines, we constructed an immune-enhancing protein that simultaneously acted as an antigen and adjuvant to enhance cellular and humoral immune responses as well as induce a broad spectrum of host defenses.

Then, we investigated the protective effect on mice against FMDV types O and A challenges using test vaccines with rpHSP70-AD and without antigens. Our results provide impactful information in terms of future FMD vaccine studies.

Commercially available FMD vaccines use oil emulsions (water-in-oil, W/O, and water-in-oil-in-water, W/O/W) as adjuvants. The induction of immune responses by non-oil emulsions or low oil containing vaccines is very difficult in target animals, especially pigs. Therefore, despite the local side effects near the vaccination site by oil emulsion, it is recognized that oil emulsion-free vaccines are virtually inapplicable.

To ameliorate the local side effects and improve safety, we evaluated early, mid-term, and long-term immune responses and protective effects in hosts induced by oil emulsion-free test vaccines containing rpHSP70-AD adjuvants. Further, to overcome the limitations of oil emulsion-free test vaccines and identify the efficacy of rpHSP70-AD adjuvant, a test vaccine containing either FMDV O or FMDV A antigen was administered to mice. Our results validated a significantly higher level of antibody titers and VN titers in the group treated with rpHSP70-AD than in the FMDV antigen only administered group (Fig. 4). Challenge experiments with FMDV O and FMDV A at 84 and 168 dpv showed 100% host defense against viral infection, indicating that strong cellular and humoral immune responses led to long-lasting memory responses in the host (Fig. 4). Therefore, oil emulsion-free test vaccines containing rpHSP70-AD adjuvants might be applied to target animals such as pigs.

Before vaccine testing in pigs, naïve porcine PBMCs were isolated from the whole blood of FMD antibody-seronegative pigs to verify rpHSP70-AD-induced cellular immune responses. The rpHSP70-AD significantly increased cell proliferation in porcine PBMCs by more than 1.6-fold compared to the NC group (without rpHSP70-AD). Consistent with the in vivo mouse study, vaccines containing rpSHP70-AD effectively induced proinflammatory and anti-inflammatory cytokine expression. Notably, high expression levels of APC-derived cytokines such as IL-1β, IL-6, and IL-12/23p40 seemed to promote the production of IL-17A by stimulating unconventional T cells such as γδ T cells and iNKT cells. These APC-derived cytokines and unconventional T cell-derived cytokines may induce synergistic effects, thereby inducing CD4^+^, CD8^+^ IL-2, and IFNγ expression. Meanwhile, the expression of anti-inflammatory cytokine IL-10 was high, which may have controlled the “cytokine storm” caused by elevated proinflammatory cytokine expression (Fig. 5).

Then, we evaluated the early, mid-term, and long-term immunity induced by an oil emulsion-free test vaccine containing rpHSP70-AD adjuvant using pigs. The antibody titers using SP A ELISA and VN titers were significantly higher in the A22 + rpHSP70-AD-treated group than in the A22 antigen-only-treated group. Contrastingly, in the group treated with the A22 antigen alone, there was little increase in antibody and VN titers despite receiving a booster vaccine 4 weeks after the first vaccination (Fig. 6). Reportedly, if the VN titer by vaccination is >2 (log_10_), FMD vaccine-induced host defense is possible^36^. Additionally, according to the FMD vaccine evaluation criteria in Korea, host defense is possible when the VN titer induced by commercial vaccination is >1.5 (log_10_). In this study, VN titers were as high as >2 (log_10_) after boosting in pigs receiving an oil emulsion-free vaccine containing rpHSP70-AD as an adjuvant and maintained up to 84 dpv (Fig. 6). Therefore, it was expected that the FMD test vaccine containing rpHSP70-AD would sufficiently protect the host. These results provided conclusive evidence that while the induction of antibody titers, VN titers, and the humoral immune response were difficult in oil emulsion-free conditions, rpHSP70-AD could be used as a highly effective novel adjuvant even in the absence of oil emulsion in vaccines.

Finally, we evaluated rpHSP70-AD-mediated host defenses against FMDV O and FMDV A challenge in pigs vaccinated with the bivalent (O + A) vaccine. The rpHSP70-AD-containing FMD vaccine induced a potent immune response, including antibody titers, and completely protected the host against FMDV infection (Fig. 7).

Thus, we propose that rpHSP70-AD, a new immunopotent recombinant protein, effectively induces a humoral immune response with a powerful cellular immune response and elicits strong early-term, mid-term, and long-term immune responses. Further, our results demonstrate that vaccines containing our novel adjuvant have a broad spectrum of protective effects in mice and pigs. Additionally, rpHSP70-AD is likely an innovative FMD vaccine adjuvant that ameliorates the local side effects caused by oil emulsion at the vaccination site in pigs. The development of a novel concept adjuvant using this strategy will provide a new approach for the design of vaccines against other viral diseases such as African swine fever and COVID-19, for which vaccine development is challenging.

## Methods

### Gene synthesis of the immune-enhanced recombinant protein

Figure 1 shows a schematic diagram of the development of rpHSP70-AD, the immune-enhancing recombinant protein. The active domain of the long-term immune-enhancing protein HSP70, the universal T cell epitope 3A, the B cell epitope of FMDV type O (O/JC/SKR/2014, GenBank: KX162590, O/TWN/97, GenBank: AY593835), the B cell epitope of FMDV type A (A/GP/2018, GenBank: MK463492, A GVII: Ban-GA, GenBank: KJ754939), the VP1 region of FMDV, the delivery molecule AP, the immune-enhancing peptide PADRE, and the universal T cell epitope Invasin were combined to produce an immunopotent recombinant gene (2396 bp) that had a broad-spectrum host defense against FMDV type O and FMDV type A. The peptide sequence “GGSGG” was inserted as a linker (Fig. 1 and Supplementary Table S1). The final synthesized rpHSP70-AD product was cloned into a pBT7-C-His vector.

### Expression of the immunopotent recombinant protein

To express the recombinant rpHSP70-AD, the amplified PCR product of rpHSP70-AD was cloned between the Nde I and Xho I restriction enzyme sites of the pET28a(+) vector using HSP70 primers. The primer sequences used are as follows: HSP70-F, TGCCGCGCGGCAGCCATATGGCACGTGCAGTTGGTATCGA; HSP70-R, TGGTGGTGGTGGTGCTCGAGAAACTGATATGTTGCGGTAT. Recombinant pET28a(+)-rpHSP70-AD plasmid was transformed into *E.coli* BL21 (DE3) cells, and a single colony of the transformed pET28a(+)-rpHSP70-AD was grown in 3 mL Luria-Bertani (LB, BD Biosciences, Franklin Lakes, NJ, USA) broth with 50 μg/mL kanamycin (Sigma-Aldrich, St. Louis, MO, USA) for 3 h at 37 °C. After inoculating the incubated cultures in 250 mL LB broth, the mixture was cultured until the OD600 reached 0.7. Then, 1 mM isopropyl β-D-1-thiogalactopyranoside (IPTG, Sigma-Aldrich) was added to induce recombinant protein expression for 12 h. Then, after recovering the *E. coli* by centrifugation at 8000 rpm, 50 mL 1× PBS (Gibco, Carlsbad, CA, USA) and 1× protease inhibitor cocktail (Roche Diagnostics, Mannheim, Germany) were added to homogenize the *E. coli* via sonication (Amp 20%, 5 s; ON, 2 s; OFF, 5 min). The homogenized *E. coli* were centrifuged at 8,000 rpm for 30 min at 4 °C in a high-speed centrifuge (Beckman Coulter, Brea, CA, USA). The collected supernatant was processed with HisPur Ni-NTA resin (Thermo Fisher Scientific, Rockford, IL, USA) to separate and purify the recombinant protein. The eluted rpHSP70-AD (15 μg) was separated via 10% sodium dodecyl sulfate-polyacrylamide gel electrophoresis (SDS-PAGE) and stained with Sun-Gel staining solution (LPS solution, Daejeon, Korea) at room temperature (RT). To confirm the purified rpHSP70-AD, SDS-PAGE was followed by western blot analysis using an anti-His antibody (R&D Systems, Minneapolis, MN, USA). All blots were derived from the same experiment and were processed in parallel.

### Purification of the antigen (inactivated virus) from O/TWN/97-R, A22/IRAQ-R, O/SKR/BE/2017, and A/SKR/YC/2017

Purified antigen (inactivated FMDV) was prepared in BHK-21 cells infected with FMDV O/TWN/97-R (GenBank AY593823), A22/IRAQ-R (GenBank AY593763), O/SKR/BE/2017 (GenBank MG983730), and A/SKR/YC/2017 (GenBank KY766148.1) as previously described^14,15^.

For the viral infection, the culture medium was replaced with serum-free Dulbecco’s modified Eagle’s medium (DMEM; HyClone, Logan, UT, USA), and the cells were inoculated with the virus by incubating for 1 h at 37 °C in 5% CO_2_ atmosphere. The extracellular viruses were then removed. Twenty-four hours post-infection, the viruses were inactivated via two treatments of 0.003 N binary ethylenimine for 24 h in a shaking incubator, followed by concentration with polyethylene glycol (PEG) 6000 (Sigma-Aldrich). The viral concentrate was layered onto a 15–45% sucrose density gradient and centrifuged.

After ultracentrifugation, the bottom of the centrifuge tube was punctured, and 1 mL fractions were collected. The presence of FMDV particles in a sample of each fraction was confirmed by measuring optical density using a lateral flow device (BioSign FMDV Ag; Princeton BioMeditech, Princeton, NJ, USA). Before using in field experiments, the PEG-pretreated supernatant was passaged through ZZ-R127 and BHK-21 cells at least twice to verify that no cytopathic effect (CPE) occurred, thereby confirming the absence of live virus in the supernatant.

### rpHSP70-AD mediated cytokine production in vivo

Age- and sex-matching wild-type C57BL/6 mice (6–7 weeks old females) were purchased from KOSA BIO Inc. (Gyeonggi, Korea). All mice were housed in micro isolator cages with ad libitum access to food and water in a specific pathogen-free biosafety level 3 (ABSL3) animal facility at the Animal and Plant Quarantine Agency. All animals were allowed to adapt for at least 1 week before use in experiments. The housing room was set to a 12/12 h light/dark cycle, a temperature of about 22 °C, and relative air humidity of about 50%. Studies were performed according to institutional guidelines and with approval from the Ethics Committee of the Animal and Plant Quarantine Agency (Accreditation number IACUC-2019–185)^14,15^.

In vivo cytokine production was evaluated via i.p. injection of mice (*n* = 5/group) with 50 μg rpHSP70-AD in 100 μL Dulbecco’s phosphate-buffered saline (DPBS; BioWhittaker-LONZA, Walkersville, MD, USA). The NC group of mice was injected with 100 μL DPBS via the same injection route. After 0, 6, 12, and 24 h, the mice were sacrificed; peritoneal lavage fluid was collected by washing the peritoneal cavity with 2 mL chilled DPBS. The peritoneal lavage fluid was centrifuged at 300 × *g* for 10 min at 4 °C, and the supernatants were stored at −80 °C for subsequent cytokine analysis.

ELISA for IL-1β, IL-2, IL-4, IL-6, IL-9, IL-10, IL-12/23p40, IL-15/15 R, IL-17A, IL-21, IL-22, IL-33, IFNγ, and TNFα (Invitrogen, Thermo Fisher Scientific Inc., Vienna, Austria; R&D Systems, Inc., Minneapolis, MN, USA) was performed on peritoneal lavage fluid supernatants. The detection limits were as follows: IL-1β, 15.6–1000 pg/mL; IL-2, 3.125–200 pg/mL; IL-4, 7.8–500 pg/mL; IL-6, 7.8–500 pg/mL; IL-9, 62.5–4000 pg/mL; IL-10, 62.5–4000 pg/mL; IL-12/23p40, 4.7–300 pg/mL; IL-15/15R, 7.8–500 pg/mL; IL-17A, 7.8–500 pg/mL; IL-21, 31.3–2000 pg/mL; IL-22, 15.6–1000 pg/mL; IL-33, 46.9–3000 pg/mL; IFNγ, 31.3–2000 pg/mL; and TNFα, 15.6–1000 pg/mL. Briefly, flat-bottom 96-well plates (Corning^TM^ Costar^TM^ 9018 ELISA plate, Corning Inc., New York, NY, USA) were coated with 100 μL/well capture antibodies in coating buffer and incubated overnight at 4 °C. The wells were aspirated and washed five times with >250 μL/well wash buffer (PBS supplemented with 0.05% (v/v) Tween 20, PBST) using a microplate dispenser (Stat-Matic II Plate Washer, Sigma-Aldrich). After patting them dry, 200 μL/well of blocking buffer was added to each well, and the plates were incubated for 1 h at RT. The plates were aspirated, washed three times again, and patted dry. One hundred microliters per well of the peritoneal lavage fluid supernatants and serially diluted standards were added to the appropriate wells, and the plates were incubated at RT for 2 h. After another washing and drying step, 100 μL/well of the biotinylated detection antibodies were added to all wells, and the plates were incubated at RT for 1 h. The wells were washed and patted dry, 100 μL/well of diluted streptavidin-horseradish peroxidase conjugate was added, and the plates were incubated at RT for 20 min. Then, the plates were washed again and dried. The peroxidase was developed with 100 μL/well of 1× TMB solution for 15 min at RT, and the reaction was stopped with 100 μL 2 N H_2_PO_4_. Absorbance was measured within 30 min using a Hidex 300SL spectrophotometer (Hidex, Finland) set at 450 nm.

### rpHSP70-AD-induced broad-spectrum host defenses in mice and early, mid-term, and long-term immune responses induced by oil emulsion-free test vaccine containing rpHSP70-AD as an adjuvant

Mouse experiments were conducted according to the method described previously in the section on rpHSP70-AD-mediated cytokine production in vivo.

The following experiment was conducted to investigate the immunogenicity of rpHSP70-AD and evaluate host defenses against FMDV (O type, A type) infection. To evaluate the immunogenicity of rpHSP70-AD, the antigens were excluded from the test vaccine, and the vaccine composition used in the experiment was as follows; without (w/o) antigen, 10 μg/dose/mouse of rpHSP70-AD, ISA 206 (Seppic, Paris, France; 50%, w/w), 10% Al(OH)_3_, and 15 μg/dose/mouse of Quil-A (InvivoGen, San Diego, CA, USA). Mice in the NC group received an equal volume of phosphate-buffered saline (PBS, pH 7.0) administered via the same route.

Mice were vaccinated via intramuscular (i.m.) injection on day 0. At 7 dpv, FMDV A (100 LD_50_ of A/Malay/97, SEA topotype) was administered via i.p. injection, and the survival rate and weight change of the mice were monitored until 7 dpc (Fig. 3a).

To evaluate the efficacy of the novel recombinant protein rpHSP70-AD as an FMD vaccine adjuvant, an oil emulsion-free test vaccine that included rpHSP70-AD as an adjuvant was produced, following the protocol published by Lee et al.^15^. Then, early, mid-term, and long-term immunity and the induced immune memory response was observed in mice. Inactivated virus isolated and purified from O/TWN/97-R (FMDV O) or A22/IRAQ-R (FMDV A) was used as the antigen. The composition of the test vaccine was as follows; FMDV O (O/TWN/97-R; O TWN) antigen or FMDV A (A22/IRAQ-R; A22) antigen (15 μg/dose/mL for cattle or pigs, 1/10 dose/100 μL for mice), rpHSP70-AD (10 μg/dose/mouse), without (w/o) oil emulsion, 10% Al(OH)_3_, and 15 μg/dose/mouse Quil-A. The positive control group was administered with a vaccine that did not include rpHSP70-AD, and the NC group was administered an equal volume of PBS (pH 7.0) via the same route. Mice (*n* = 5/group) were first vaccinated with the test vaccine via the i.m. route and were boosted via the same route at 35 dpv. After primary vaccination, blood was collected on 0, 7, 14, 28, 56, 84, and 168 dpv and used for serological analysis such as SP A ELISA and VN titers. The mice were challenged with FMDV (100 LD_50_ of O/VET/2013, ME-SA topotype or A/Malay/97, Asia topotype) via an i.p. injection on 84 and 168 dpv. Survival rate and weight changes were monitored until 7 dpc (Fig. 4a).

### rpHSP70-AD-mediated cellular immune responses in PBMCs

Porcine PBMCs were isolated from the whole blood of FMD antibody-seronegative pigs as donors (10–12 weeks old animals, *n* = 3/group) according to the method described by Lee et al.^14^. Whole blood (20 mL/donor) was independently collected in BD Vacutainer heparin tubes (BD, Becton, Dickinson and Company, Franklin Lakes, NJ, USA). PBMCs were isolated using Ficoll-Paque PLUS (GE Healthcare Bio-Sciences Corp., Piscataway, NJ, USA) gradient centrifugation. Residual red blood cells were lysed with ammonium-chloride-potassium (ACK) lysing buffer (Gibco, Carlsbad, CA, USA). PBMCs were suspended in Ca^2+^ and Mg^2+^-free DPBS (Gibco) supplemented with 2% fetal bovine serum (FBS) (Gibco) and counted using a volumetric flow cytometer (Miltenyi Biotec, Bergisch Gladbach, Germany). All cells were freshly isolated before use. No cryopreserved cells were used in any experiment. Purified PBMCs were then resuspended in RPMI-1640 (Gibco) medium supplemented with 10% FBS (HyClone, Logan, UT, USA), 3 mM L-glutamine (Sigma-Aldrich), and 100 U/mL penicillin–streptomycin (Sigma-Aldrich).

Isolated porcine (*n* = 3) PBMCs were treated with or without 2 μg/mL rpHSP70-AD. After 24 h, the cell culture medium (supernatant) was harvested for ELISA, and cell proliferation was assessed via 5-bromo-2′-deoxyuridine (BrdU) incorporation.

The effects of rpHSP70-AD on the proliferation of porcine PBMCs were assessed using BrdU Cell Proliferation Assay Kit (Cell Signaling Technology, Beverly, MA, USA) based on the incorporation of BrdU during DNA synthesis. Briefly, 10 µM BrdU was added to the cell culture medium. Then, the cells were incubated for 16 h at 37 °C. The cells were then fixed and incubated with anti-BrdU mouse monoclonal antibody, followed by horseradish peroxidase-conjugated goat anti-mouse antibodies. The chromogenic substrate, tetramethylbenzidine (TMB), was used for color development. Absorbance was measured within 30 min using a Hidex 300SL spectrophotometer (Hidex, Finland) set at 450 nm^14^.

ELISAs for porcine IL-1β, IL-2, IL-6, IL-10, IL-12/23p40, IL-17A, and IFNγ (DuoSet, R&D Systems, Minneapolis, MN, USA; KingFisher Biotech, Inc., St. Paul, MN, USA) were performed on cell culture supernatants. The detection limits were as follows: IL-1β, 62.5–4000 pg/mL; IL-2, 46.9–3000 pg/mL; IL-6, 12.5–8000 pg/mL; IL-10, 23.4–1500 pg/mL; IL-12/23p40, 78.1–5000 pg/mL; IL-17A, 0.8–25 ng/mL; and IFNγ, 62.5–4000 pg/mL. Cytokine ELISA was performed according to the method previously mentioned in the section on the rpHSP70-AD-mediated cytokine production in vivo (assessment of pro- and anti-inflammatory cytokine production using ELISA).

### Early, mid-term, and long-term immune response of oil emulsion-free test vaccines including rpHSP70-AD as an adjuvant in pigs

To evaluate the potential of rpHSP70-AD as an FMDV vaccine adjuvant, especially as an immunostimulant, and investigate its ability to induce cellular and humoral immune responses and long-term immunity, target animal experiments using pigs were performed.

For the target animal experiments, FMD type A antibody-seronegative animals were used (the pigs were 10–12 weeks old). The pigs were divided into two groups (*n* = 5/group) (Fig. 6a).

The animals were isolated in closed containments (Animal Biosafety Level 3, ABSL-3) at the Animal and Plant Quarantine Agency during the study. After arrival at our ABSL facility, all animals were kept in cages with ad libitum access to food and water and used for experiments after at least 1 week of adaptation. The housing room was set to a 12/12 h light/dark cycle, a temperature of about 22 °C, and relative air humidity of about 50%. The studies were performed according to institutional guidelines, with approval from the Ethics Committee of the Animal and Plant Quarantine Agency (Accreditation number IACUC-2019–185).

The composition of the oil emulsion-free test vaccine including rpHSP70-AD as an adjuvant to evaluate the induction of early, mid-term, and long-term immunity in pigs was as follows: FMDV A (A22) antigen (15 μg/dose/pig/mL), without rpHSP70-AD (positive control group) or with rpHSP70-AD (experimental group), without emulsion, with 10% Al(OH)_3_, and 150 μg/dose/pig Quil-A (InvivoGen). One milliliter vaccine was prepared as a single dose.

After primary vaccination via i.m. injection, a booster dose was injected at 28 dpv via the same route. Blood was collected at 0, 7, 14, 28, 42, 56, 70, and 84 dpv to observe the antibody titer via SP ELISA and VN titer (Fig. 6a). The animals were monitored daily for body temperature, symptoms at the vaccination site, and appetite. Serum samples were stored at −80 °C until tests were performed.

### Host defense in response to oil emulsion-free test vaccines including rpHSP70-AD as an adjuvant in pigs against FMDV type O and FMDV type A infection

To evaluate the effect of rpHSP70-AD-mediated host defense in pigs, a challenge experiment in target animals using pigs was performed.

For this study, FMD type O and FMD type A antibody-seronegative animals were used (the pigs were 10–12 weeks old), and the pigs were divided into six groups (*n* = 3 or 5/group) (Fig. 7a). The study in ABSL3 at the Animal and Plant Quarantine Agency was performed according to the above-mentioned condition and institutional guidelines.

Pigs were immunized with a single dose of oil emulsion-free bivalent (O + A) test vaccine with or without rpHSP70-AD as an adjuvant to evaluate the protective effect against FMDV infection.

The composition of the oil emulsion-free bivalent test vaccine including rpHSP70-AD as an adjuvant was as follows: FMDV O (O/SKR/BE/2017; O BE, 15 μg/dose/pig/mL) and FMDV A (A/SKR/YC/2017; A YC, 15 μg/dose/pig/mL) antigen, without rpHSP70-AD (positive control group) or with rpHSP70-AD (experimental group), without emulsion, with 10% Al(OH)_3_, and 150 μg/dose/pig Quil-A (InvivoGen). One milliliter vaccine was prepared as a single dose and introduced into the animals via i.m. injection. The NC group of pigs was treated with an equal volume of PBS via the same route. After single-shot vaccination at 0 dpv, blood was collected at 0 and 28 dpv to observe the antibody titer via SP ELISA and VN titer (Fig. 7a). The body temperature, symptoms at the vaccination site, and appetite were monitored daily. Serum samples were stored at −80 °C until the tests were performed.

Vaccinated pigs were challenged with FMDV type O (O/SKR/JC/2014, Asia topotype) or FMDV type A (A/SKR/GP/2018, Asia topotype) via i.d. (intradermal) injection on the heel bulb of pigs at 10^5^ TCID_50_/100 μL. Clinical symptoms and body temperature were monitored daily from challenged pigs from 0 to 8 dpc. Sera samples were collected on 0, 2, 4, 6, and 8 dpc by venipuncture (anterior vena cava) and transferred into Vacutainer serum tubes (BD Biosciences, San Jose, CA, USA). Oral swab samples were collected daily from 0 to 8 dpc using the BD^TM^ Universal Viral Transport Kit (BD Biosciences).

The clinical score was confirmed as follows: (1) An elevated body temperature of 40 °C (1 point), >40.5 °C (2 points), or >41 °C (3 points); (2) reduced appetite (1 point) or no food intake and food leftover from the day before (2 points); (3) lameness (1 point) or reluctance to stand (2 points); (4) the presence of heat and pain after palpation of the coronary band (1 point) or not standing on the affected foot (2 points); (5) vesicles on the feet, dependent on the number of feet affected, with a maximum of 4 points; (6) visible mouth lesions on the tongue (1 point), gums or lips (1 point), or snout (1 point), with a maximum of 3 points^37^.

For viral load assay, viral RNAs were extracted from the sera and swab samples using QIAcube® HT Pathogen Kit (QIAGEN, Leipzig, Germany); then, RT-PCR was performed using the one-step prime-script RT-PCR kit (Bioneer, Korea). The following primer pair targeting the FMDV 3D region was used: sense primer, 5′–GGAACYGGGTTTTAYAAACCTGTRAT–3′; antisense primer 5′–CCTCTCCTTTGCACGCCGTGGGA–3′. The probe with the following sequence, 5′–CCCADCGCAGGTAAAGYGATCTGTA–3′, was labeled with 6-FAM at the 5′ end and with TAMRA at the 3′ end. The CFX96 Touch^TM^ Real-Time PCR Detection System (Bio-Rad, Hercules, CA, USA) was used for virus quantification.

### Serological assays

To detect SP antibodies in the sera, PrioCHECK^TM^ FMDV type O or FMDV type A (Prionics AG, Switzerland) and VDPro^®^ FMDV type O or FMDV type A (Median Diagnostics, Gangwon-do, Korea) was used. Absorbance in the ELISA plate was converted to percent inhibition (PI) value. When the PI value was 50% for the PrioCHECK^TM^ FMDV kit or 40% or above for the VDPro^®^ FMDV kit, the animals were considered antibody positive.

A virus neutralization test (VNT) was performed according to the World Organization for Animal Health (OIE) manual^3,14,15^. The sera were heat-inactivated at 56 °C for 30 min in a water bath. Cell density was adjusted to form a 70% monolayer, and 2-fold serial dilutions of sera samples (1:8–1:1024) were prepared. The diluted sera samples were then incubated with a 100-tissue culture infectious dose (TCID)_50_/0.5 mL homologous virus for 1 h at 37 °C. After 1 h, LF-BK (bovine kidney) cell suspension was added to all wells. After 2–3 days, CPE was evaluated to determine the titers, which were calculated as log_10_ of the reciprocal antibody dilution required to neutralize 100 TCID_50_ of the virus^38,39^. FMDV A22/IRAQ, FMDV O/SKR/BE/2017, and FMDV A/SKR/YC/2017 were used for VNT.

### Statistics

All quantitative data were expressed as the mean ± standard error (SEM) unless otherwise stated. Between groups, statistical significance was assessed using two-way ANOVA followed by Bonferroni post hoc test or one-way ANOVA followed by Tukey’s post hoc test. ^∗^*p* < 0.05; ^∗∗^*p* < 0.01; and ^∗∗∗^*p* < 0.001. Parametric tests were used to compare different groups. Survival curves were built using the Kaplan-Meier method, and differences were analyzed using the log-rank sum test. GraphPad Prism 5 (GraphPad, San Diego, CA, USA) software and IBM SPSS software (IBM Corp., Armonk, NY, USA) were used for all statistical analyses.

### Reporting summary

Further information on the experimental design is available in the Nature Research Reporting Summary linked to this article.

## Supplementary information

Supplementary Information

Reporting Summary

## Data Availability

All data that support the findings of this study are available from the corresponding author upon reasonable request.
