# Supplementary material for: The HSP70-fused foot-and-mouth disease epitope elicits cellular and humoral immunity and drives broad-spectrum protective efficacy
Source: NPJ Vaccines. 2021 Mar 26;6:42. doi: 10.1038/s41541-021-00304-9 (PMC7998017; doi:10.1038/s41541-021-00304-9)
Supplement: Supplementary file 1 — Supplementary Information [file 41541_2021_304_MOESM1_ESM.pdf]

Supplementary information

Supplementary Table 1. rpHSP70-AD sequence information

Supplementary Figure 1. Full size image of Figure 1b and 1c

Supplementary Figure 2. rpHSP70-AD-mediated host defense against FMDV type O and type A infection and changes in body temperature

Supplementary Table 1

rpHSP70

1	ARAVGIDLGT	TNSVVSVLEG	GDPVVVANSE	GSRTTPSIVA	FARNGEVLVG	QPAKNQAVTN	60
61	VDRTVRSVKR	HMGSDWSIEI	DGKKYTAPEI	SARILMKLKR	DAEAYLGEDI	TDAVITTPAY	120
121	FNDAQRQATK	DAGQIAGLNV	LRIVNEPTAA	ALAYGLDKGE	KEQRILVFDL	GGGTFDVSLL	181
181	EIGEGVVEVR	ATSGDNHLGG	DDWDQRVVDW	LVDKFKGTSG	IDLTKDKMAM	QRLREAAEKA	240
241	KIELSSSQST	SINLPYITVD	ADKNPLFLDE	QLTRAEFQRI	TQDLLDRTRK	PFQSVIADTG	300
301	ISVSEIDHVV	LVGGSTRMPA	VTDLVKELTG	GKEPNKGVNP	DEVVAVGAAL	QAGVLKGEVK	360
361	DVLLLDVTPL	SLGIETKGGV	MTRLIERNTT	IPTKRSETFT	TADDNQPSVQ	IQVYQGEREI	420
421	AAHNKLLGSF	ELTGIPPAPR	GIPQIEVTFD	IDANGIVHVT	AKDKGTGKEN	TIRIQEGSGL	480
481	SKEDIDRMIK	DAEAHAEEDR	KRREEADVRN	QAETLVYQTE	KFVKEQREAE	GGSKVPEDTL	540
541	NKVDAAVAEA	KAALGGSDIS	AIKSAMEKLG	QESQALGQAI	YEAAQAASQA	TGAAHPGGEP	600
601	GGAHPGSADD	VVDAEVVDDG	REAKGGSGGA	AIEFFEGMVH	DSIKGGSGGL	PNARGDLQVL	660
661	APKAARPLPN	NVRGDLQVLA	QKTEKTLPGG	SGGPQNRRGD	SGPLVVKPTQ	LPSGRVRGDL	720
721	GGLAARVAAQ	LPGGSGGHKQ	KIVAPVKQSL	RRRWCKRRRG	GSGGAKFVAA	WTLKAAAGGS	780
781	GGTAKSKKFP	S Y T A T Y Q F					798

*3A (T cell epitope); 629-644
*O/JC/2014 (B cell epitope); 649-668
*O/TWN/97 (B cell epitope); 669-688
*A/GP/2018 (B cell epitope); 694-712
*A/GVII:BAN-GA (B cell epitope); 713-712
*VP1 (200-213 bp); 738-750
*AP (Delivery molecule); 751-759
*PADRE (Immunopotent molecule); 765-777
*Invasin (T cell epitope); 783-798
*Linker; GGSGG

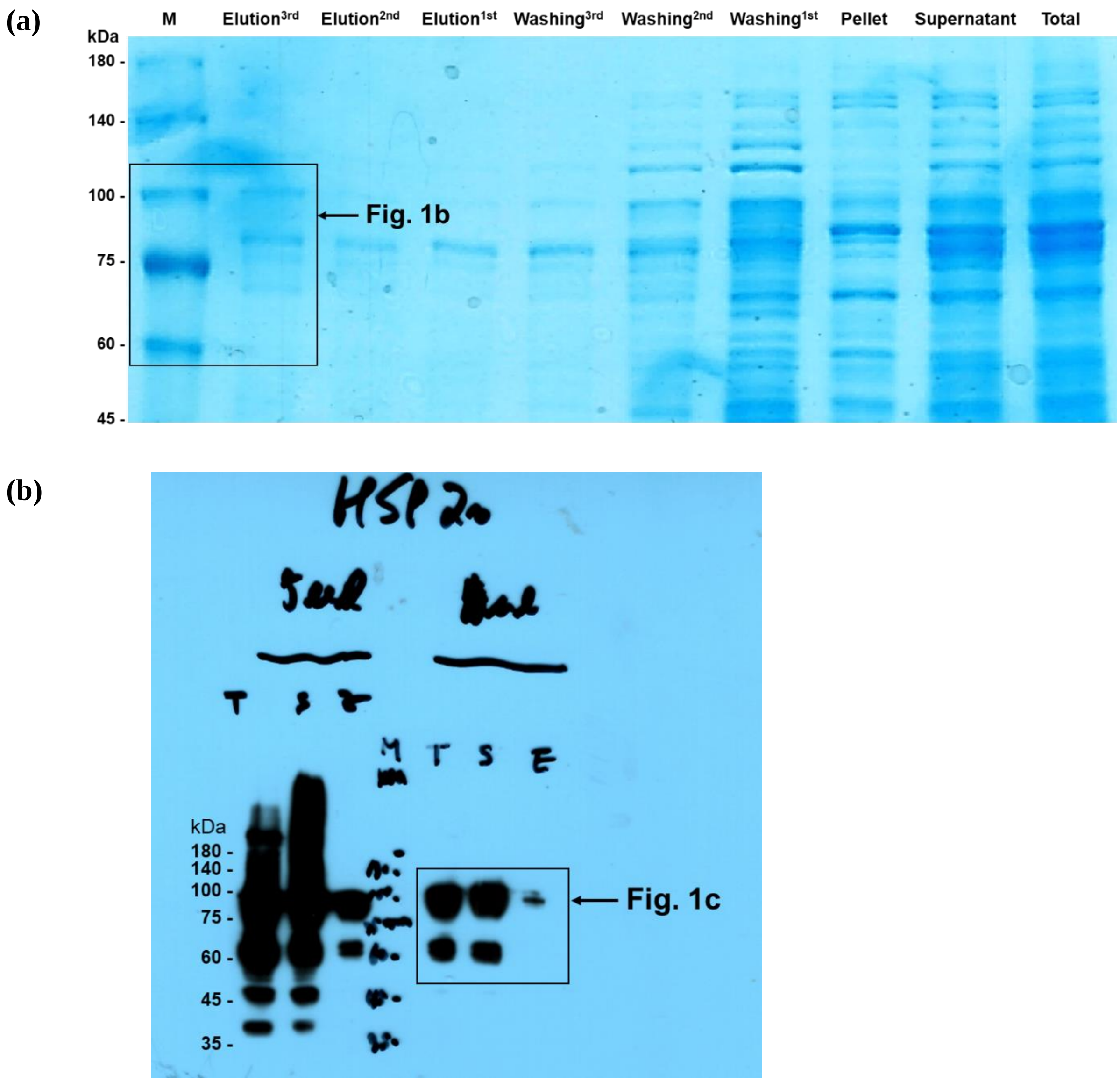

Supplementary Figure 1

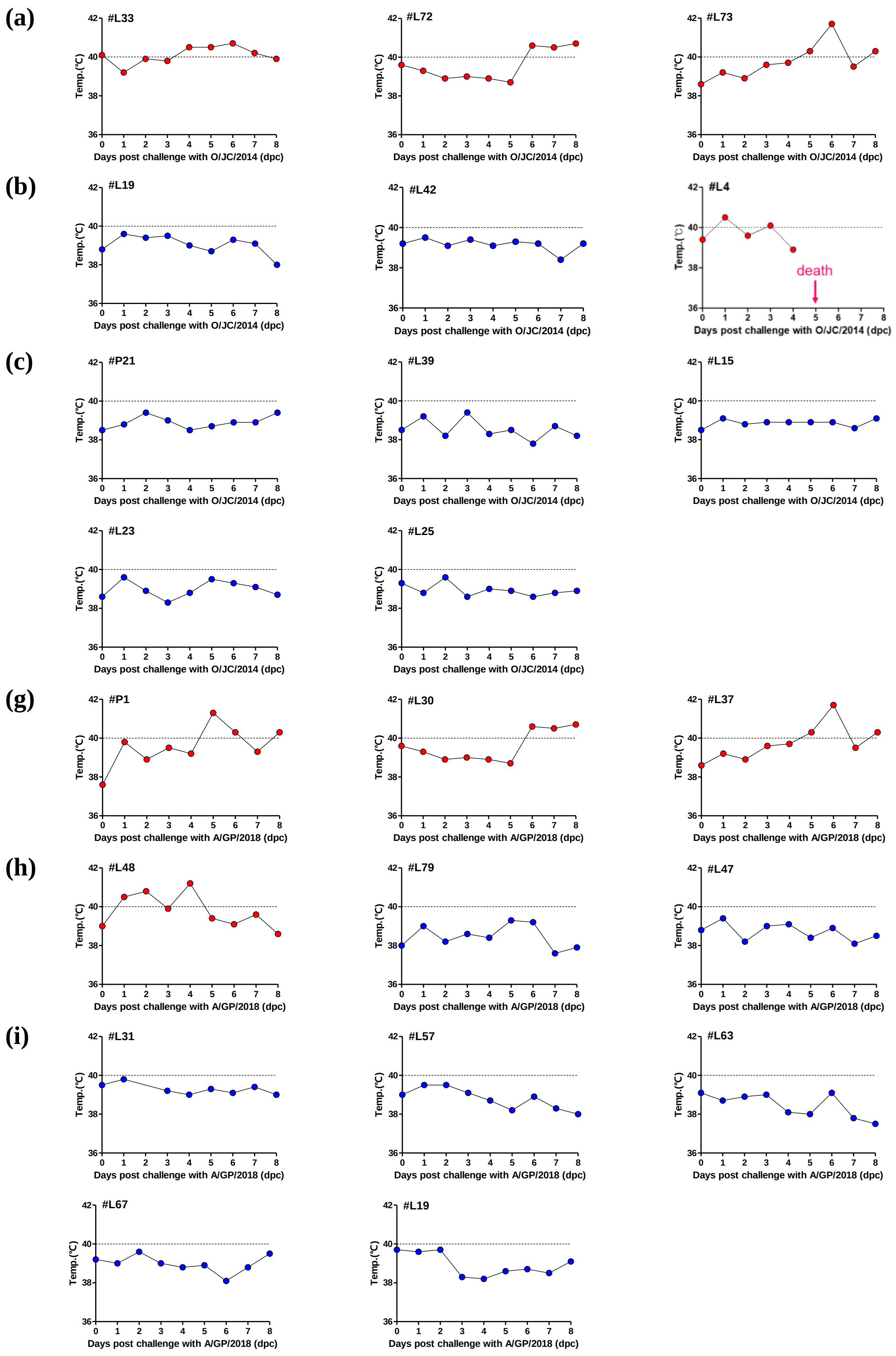

Supplementary Figure 2
