# Supplementary material for: The HSP70-fused foot-and-mouth disease epitope elicits cellular and humoral immunity and drives broad-spectrum protective efficacy
Source: NPJ Vaccines. 2021 Mar 26;6:42. doi: 10.1038/s41541-021-00304-9 (PMC7998017; doi:10.1038/s41541-021-00304-9)
Supplement: Supplementary file 2 — Reporting Summary [file 41541_2021_304_MOESM2_ESM.pdf]

nature research

Corresponding author(s): Min Ja Lee
Jong-Hyeon Park

Last updated by author(s): Feb 11, 2021

Reporting Summary

Nature Research wishes to improve the reproducibility of the work that we publish. This form provides structure for consistency and transparency in reporting. For further information on Nature Research policies, see our Editorial Policies and the Editorial Policy Checklist.

Statistics

For all statistical analyses, confirm that the following items are present in the figure legend, table legend, main text, or Methods section.

n/a	Confirmed	
☐	☒	The exact sample size (n) for each experimental group/condition, given as a discrete number and unit of measurement
☐	☒	A statement on whether measurements were taken from distinct samples or whether the same sample was measured repeatedly
☐	☒	The statistical test(s) used AND whether they are one- or two-sided<br>*Only common tests should be described solely by name; describe more complex techniques in the Methods section.*
☐	☒	A description of all covariates tested
☐	☒	A description of any assumptions or corrections, such as tests of normality and adjustment for multiple comparisons
☐	☒	A full description of the statistical parameters including central tendency (e.g. means) or other basic estimates (e.g. regression coefficient) AND variation (e.g. standard deviation) or associated estimates of uncertainty (e.g. confidence intervals)
☐	☒	For null hypothesis testing, the test statistic (e.g. F, t, r) with confidence intervals, effect sizes, degrees of freedom and P value noted<br>*Give P values as exact values whenever suitable.*
☒	☐	For Bayesian analysis, information on the choice of priors and Markov chain Monte Carlo settings
☒	☐	For hierarchical and complex designs, identification of the appropriate level for tests and full reporting of outcomes
☒	☐	Estimates of effect sizes (e.g. Cohen's d, Pearson's r), indicating how they were calculated

Our web collection on statistics for biologists contains articles on many of the points above.

Software and code

Policy information about availability of computer code

Data collection	No software was used to collect data in the study.
Data analysis	All software used is freely available.

For manuscripts utilizing custom algorithms or software that are central to the research but not yet described in published literature, software must be made available to editors and reviewers. We strongly encourage code deposition in a community repository (e.g. GitHub). See the Nature Research guidelines for submitting code & software for further information.

Data

Policy information about availability of data

All manuscripts must include a data availability statement. This statement should provide the following information, where applicable:

- Accession codes, unique identifiers, or web links for publicly available datasets
- A list of figures that have associated raw data
- A description of any restrictions on data availability

All data generated or analyzed during this study are included in this published article (and its supplementary information files).
The authors declare that all other data supporting the findings of this study are available within the article and its Supplementary Information files, or are available from the authors upon request.

Field-specific reporting

Please select the one below that is the best fit for your research. If you are not sure, read the appropriate sections before making your selection.

☒ Life sciences ☐ Behavioural & social sciences ☐ Ecological, evolutionary & environmental sciences

For a reference copy of the document with all sections, see nature.com/documents/nr-reporting-summary-flat.pdf

Life sciences study design

All studies must disclose on these points even when the disclosure is negative.

Sample size	All assays were repeated as multiple independent experiments as stated in the individual figure legends.
Data exclusions	No data were excluded.
Replication	All experiments were repeated multiple times as stated in the in the individual figure legends.
Randomization	Mice and pigs were age and gender matched; they were randomized and distributed equally into experimental groups.
Blinding	No blinding was used. There were no subjective measurements requiring blinded analysis.

Reporting for specific materials, systems and methods

We require information from authors about some types of materials, experimental systems and methods used in many studies. Here, indicate whether each material, system or method listed is relevant to your study. If you are not sure if a list item applies to your research, read the appropriate section before selecting a response.

Materials & experimental systems

n/a	Involved in the study	
☐	☒	Antibodies
☐	☒	Eukaryotic cell lines
☒	☐	Palaeontology and archaeology
☐	☒	Animals and other organisms
☒	☐	Human research participants
☒	☐	Clinical data
☒	☐	Dual use research of concern

Methods

n/a	Involved in the study	
☒	☐	ChIP-seq
☒	☐	Flow cytometry
☒	☐	MRI-based neuroimaging

Antibodies

Antibodies used	See method section of the manuscript.
Validation	See method section of the manuscript.

Eukaryotic cell lines

Policy information about cell lines

Cell line source(s)	See method section of the manuscript.
Authentication	No authentication of cell lines was conducted.
Mycoplasma contamination	All the cell lines were confirmed as mycoplasma free.
Commonly misidentified lines (See ICLAC register)	None of the cell lines used are listed in the ICLAC database.

Animals and other organisms

Policy information about studies involving animals; ARRIVE guidelines recommended for reporting animal research

Laboratory animals	C57BL/6N mice (n=5/group) and Landrace pigs (n=3-5/group)were used. For most of experiments, 6-7-weeks old female mice and 10-12-weeks old female pigs were used unless otherwise noted.

Wild animals	No wild animals were used in this study.
Field-collected samples	10-12-weeks old female Landrace pigs were used in field study. The pigs were divided into each groups (n=5/group). The animals were kept in in a contaminant-free space during the study. Porcine PBMC for this study were purified from the whole blood of each pig (n=3/group).
Ethics oversight	All animal experiments were performed according to institutional guidelines with approval from the Ethics Committee of Animal and Plant Quarantine Agency (Accreditation number IACUS-2019-185).

Note that full information on the approval of the study protocol must also be provided in the manuscript.
